# Multivariate-based classification of predicting cooking quality ideotypes in rice (*Oryza sativa* L.) indica germplasm

**DOI:** 10.1186/s12284-018-0245-y

**Published:** 2018-10-10

**Authors:** Rosa Paula O. Cuevas, Cyril John Domingo, Nese Sreenivasulu

**Affiliations:** 1 International Rice Research Institute (IRRI), College, Los Baños, Laguna Philippines; 2 Present address: Pangasinan State University, Bayambang, Pangasinan Philippines

**Keywords:** Grain quality, rapid Visco-analysis (RVA), random forest model, Rheology, Texture profile analysis

## Abstract

**Background:**

For predicting texture suited for South and South East Asia, most of the breeding programs tend to focus on developing rice varieties with intermediate to high amylose content in indica subspecies. However, varieties within the high amylose content class may still be distinguishable by consumers, who are able to distinguish texture that cannot be differentiated by proxy cooking quality indicators.

**Results:**

This study explored a suite of assays to capture viscosity, rheometric, and mechanical texture parameters for characterising cooked rice texture in a set of 211 rice accessions from a diversity panel and employed multivariate approaches to classify rice varieties into distinct cooking quality classes. Results suggest that when the amylose content range is narrowed to the intermediate to high classes, parameters determined by rheometry and RVA become diagnostic. Modeled parameters distinguishing cooking quality ideotypes within the same range of amylose classes differ in textural parameters scored by a descriptive sensory panel.

**Conclusions:**

Our results reinforced the notion that it is important to define cooking quality classes in indica subtypes based on multidimensional parameters, by going beyond amylose predictions. These predictive cooking models will be handy in capturing cooking and eating quality properties that address consumer preferences in future breeding programs. Policy implications of such findings may lead to changes in criteria used in assessing grain quality in the intermediate to high amylose classes.

**Electronic supplementary material:**

The online version of this article (10.1186/s12284-018-0245-y) contains supplementary material, which is available to authorized users.

## Background

In rice varietal improvement programs, the texture of cooked rice is primarily indicated by amylose content (AC) [Juliano 2006; Juliano et al. 2009]. This parameter is used to classify rice into five AC classes associated with cooked rice texture: waxy (0–2%), very low (3–9%), low (10–19%), intermediate (20–25%), and high (> 25%) [Kumar and Khush 1986a; Kumar and Khush 1986b]. However, samples in the same AC class could have different sensory profiles [Champagne et al. 2010], which suggest that within an AC class, rice varieties are still quite diverse in terms of cooking and eating quality. Attempts to fine-tune rice characterisation include introducing gel consistency (GC) data to differentiate high-AC rice into soft and hard classes [Cagampang et al. 1973]; and gelatinisation temperature (GT) to further differentiate samples within an AC class into different GT classes [e.g.,Yang et al. 2014; Pang et al. 2016]. Hence, rice variety development and improvement programs use these AC, GT, and GC indicators to develop breeding targets for specific markets. Other attributes, such as pasting properties and mechanical textural properties of rice varieties, also provide further insights into cooked rice texture. However, many of the findings still point to associations of texture with AC [e.g., Li et al. 2017; Hori et al. 2016; Tran et al. 2011; Li et al. 2016]; thereby effectively masking associations among these attributes with the diversity of rice germplasm within an AC class. It must be noted that many of the past studies used narrow ranges of germplasm to perform associations within each AC class [e.g.,Yang et al. 2014; Tuaño et al. 2014; Garcia et al. 2011].

One of the best ways to determine the associations of other cooking quality factors with texture is to make AC a constant in studies. An approach is to focus analyses on waxy rice varieties, which have negligible concentrations of amylose. However, the global waxy rice market is small (only 1% of the rice trade); thus the waxy rice approach is not widely used [Calpe 2004]. The biggest market share for rice comes from those who prefer varieties with intermediate to high AC in South Asia and South East Asia [Tuaño et al. 2016; Calingacion et al. 2014]. Hence, insights on diversity of textural attributes would be most valuable from studies that focused on varieties coming from these two AC classes.

Information about other cooking quality attributes within an AC class may be obtained by further characterising the behaviour of starch during the cooking and the cooling process. Rapid Visco-Analysis (RVA) is routinely used to determine the pasting behaviour of starch-water suspensions. The pasting curve provides metrics that indicate disintegration and retrogradation of starch, and is based on rheological principles [Zaidul et al. 2007; Fitzgerald et al. 2003; Doutch et al. 2012]. These metrics include *peak viscosity* (PV, the maximum viscosity registered during the heating–holding stages), *trough viscosity* (TV, the minimum viscosity after PV), *final viscosity* (FV, the viscosity measurement at the end of the cooling stage), *breakdown* (BD, the difference between PV and TV), *lift-off* (LO, the difference between TV and FV), and *setback* (SB, the difference between FV and PV). The pasting curve also shows the *pasting temperature* (PTemp, temperature at the point at which the viscosity increase is greater than the set point for viscosity change rate) and the *peak time* (PT, the time it took to reach PV) [Fitzgerald et al. 2003; Bao 2008]. On the other hand, reports of viscoelastic properties of starch pastes via rheometry typically mention the values for maximum storage modulus (*G’*_max_) and feature the curves for G’, loss modulus (G”), and tan (δ) [Hsu et al. 2000; Iturriaga et al. 2006; Tsai and Lii 2000]. Extracting more information from the viscoelastic curves could provide information about the diversity of rice varieties within the same AC class.

Texture parameters could be classified into three types: geometrical properties, mechanical properties, and properties related to moisture and fat content [Brandt et al. 1963]. Texture profiling using the Texture Analyser focuses on mechanical attributes. For cooked rice, four mechanical attributes measured by the Texture Analyser are applicable: hardness, adhesiveness, cohesiveness, and springiness [Champagne et al. 1998]. These mechanical attributes could provide more dimensions in rice characterisation within the same AC class.

Classification of diverse germplasm within intermediate and high AC classes based on multidimensional data such as those generated by RVA, rheometry, and texture analysis will shed interesting insights about cooking and eating quality. Such insights could then be obtained through dimension reduction and correlation studies applied to multidimensional cooking quality data. Predicted cooking quality classes can also be used to determine grain quality attributes that affect the market prices of rice varieties [e.g., Cuevas et al. 2016; Unnevehr et al. 1985].

In this study, multinomial logistic regression and random forests are the data mining techniques that were employed. The multinomial logistic regression is an extension of the binary logistic regression, a technique used to calculate the probability of membership in one of more than two nominal or unordered categories (outcome variables) based on maximum likelihood estimation [Dixit et al. 2015, reviewed in Madhu et al. 2014]. Random forests, on the other hand, is a prediction tool composed of a combination of decision trees that can be used for classification and to measure the importance of variables [Breiman 2001]. The importance of a variable is estimated based on increases in prediction error when the data for that variable is permuted while the data for other variables are kept unchanged [Liaw and Wiener 2002]. In other words, an important variable that has a considerable effect on the accuracy of classification can be identified through a random forest model [Ziegler and König 2014].

To the best of our knowledge, these data mining techniques have not been applied to classify rice accessions based on cooking and organoleptic attributes. The objectives of this study, therefore, were to (1) characterise the textural and cooking properties of a collection of rice varieties belonging to the intermediate- and to the high-AC classes; and (2) apply modeling techniques to predict distinct cooking quality ideotypes based on visco-elastic and textural attributes.

## Methods

### Rice varieties

A set (*n* = 211) of indica rice accessions was selected based on genetic diversity and their geographic distribution, listed as Additional file 1: Table S4. These varieties were planted and grown during the dry season of 2014 under field conditions at IRRI by following the standard agronomic practices. The paddy grains were harvested at maturity and the samples were then stored to equilibrate moisture content to 14%. The samples were then dehulled (Rice sheller THU-35A, Satake Corporation, Hiroshima, Japan) and milled (Grainman 60–230-60-2AT, Grain Machinery Mfg. Corp., Miami, USA). A test portion (100 unbroken grains) from each sample was set aside for texture profile analyses (TPA) and the rest of the sample was ground to fine powder (Cyclone Sample Mill 3010–030, Udy Corporation, Fort Collins, USA). The homogenized rice flour was used for various biochemical analyses.

### Amylose content measurement

Amylose content (AC) was determined based on the colorimetric reaction of the amylose-iodine complex developed using the method of ISO 6647 [International Organization for Standardization 2007a, 2007b]. In brief, 100 mg flour was suspended in ethanol (1 mL) and sodium hydroxide (9 mL, 1 N). The suspension was then heated (95 °C, 10 min) to gelatinise the starch. Then, the sample was cooled to room temperature and the volume of the suspension was made up to 100 mL using deionised water. The starch in the gelatinised sample was injected into the glass transition lines of a San ++ Segmented Flow Analyser (SFA) system (Skalar Analytical B.V., AA Breda, The Netherlands); it was allowed to react with an aqueous solution containing 10% CH_3_COOH (1 N) and 30% KI-I_2_ (2%:0.2%) to form amylose-iodine complex. Absorbance of the sample’s amylose-iodine complex was measured at a wavelength of 620 nm and AC was quantified from a standard curve using varieties of known ACs (IR65, IR24, IR64, and IR8). The ACs of the varieties used in the standard curve were determined using the reference method of ISO 6647 [International Organization for Standardization 2007a, 2007b]. Samples were then classified into AC classes using the AC ranges previously reported [Graham 2002].

### Gelatinisation temperature (GT) measurement

The GT of the rice samples were characterised through differential scanning calorimetry (DSC) as previously described [Cuevas et al. 2010]. In brief, milled rice flour (4 mg) was immersed in water (8 mg) and the suspension was hermetically sealed in aluminum pans. The sealed pans were then heated (25–120 °C), with temperature being increased at a rate of 10 °C min^− 1^, using a DSC model Q100 (TA Instruments, DE, USA). Data on thermal transitions were collected and analysed using the Universal Analysis 2000 software. The peak of the endotherm was reported as the GT. Samples were classified as low-GT (below 67 °C), as intermediate-GT (68–73 °C), and as high-GT (GT ≥ 74 °C) [Cuevas et al. 2010; Musyoki et al. 2015].

### Rapid Visco-analyses

Rice flour (3 g) was suspended in reverse osmosis-purified (RO) water (25 g) in a canister and viscosity changes were then measured using a Rapid Visco Analyzer (RVA, Model 4-D, Newport Scientific, Warriewood, Australia), following the heat (50–95 °C) – hold (95 °C) – cool (95–50 °C) time/temperature profile described in the AACC Method 61–02 [American Association of Cereal Chemists Inc 2000]. The time/temperature profile was controlled and the data was collected and processed using the ThermoCline for Windows (TCW) version 2.6.

### Rheometry

For each sample, rice flour was suspended in reverse-osmosis water (1:2 (*w*/*v*)) and then placed at the centre of a Peltier plate of the Advanced Rheometer 2000 (TA Instruments, New Castle, DE). The rheometer was fitted with a parallel plate geometry (Ø = 40 mm). To minimise evaporation during the test, the sample was covered with a solvent trap sealed with water. During the test, the sample was subjected to heating ramp (35–95 °C) at 4 °C min^− 1^ ramp rate then to a cooling ramp (95–35 °C) at the same ramp rate. The frequency of oscillation was set at 1 cycle per second (1 Hz). Measurements were performed in triplicate. The TA Advantage Software 2003 (version 4.0.0) was used to record the data (Table 1). Additional parameters were calculated using Microsoft Excel 2013 (Table 1, Additional file 2: Figure S1). The temperature at the gelation point (i.e., the crossover point where tan (δ) = 1), the loss modulus (*G”*) at *G’*_max_, the tan (δ) at *G’*_max_ (the ratio of *G”* to *G’* at *G’*_max_), and the temperature at *G’*_max_ were determined based on the G’ and the G” curves. Slopes 1 and 3 (S1 and S3) were measured from the gel point (where tan (δ) = 1) to *G’*_max_ and *G”*_max_. Slope 2 (S2) was measured at *G’*_max_ to the lowest point of the decreasing *G’* (*G’*_trough_) while Slope 4 (S4) was measured from *G”*_max_ to the point before the *G”* leveled off.

**Table 1:** Description of the viscoelastic properties measured via rheometry [Hsu et al. 2000; Ahmed et al. 2008; Mandala 2012]

Parameter	Description
Storage Modulus max (*G’*_max_)	Maximum energy stored was reached.
Loss modulus (*G”*) at *G’*_max_	Energy loss at *G’*_max_.
Tan (δ) at *G’*_max_	Variable that describes behavior of the sample (solid- or liquid- like).
Temperature at gelation point (tan (δ) =1)	Temperature measured at point where G’ and G” crossed over and the point at which tan (δ) =1.
Peak temperature *G’*	Temperature measured when the *G’*_max_ is reached.
^a^Slope 1 (S1)	Rate of change of *G’* from the gelation point to *G’*_max_.
^a^Slope 2 (S2)	Rate of change of *G’* from *G’*_max_ to the lowest point of the decreasing *G’* (P1)
^a^Slope 3 (S3)	Rate of change of *G”* from the gelation point to *G”*_max_.
^a^Slope 4 (S4)	Rate of change of *G”* from *G”*_max_ to the point before the *G”* levels off.
^a^ *G’*_trough_	Lowest point after *G’*_max_.

^a^features of the viscoelastic curves that are not routinely measured, according to literature

### Texture profile analyses

For each sample, 25 unbroken milled rice grains were submerged in 1 mL water for 15 min in a test tube, which was then covered to minimise water evaporation. The test tube was heated in a boiling water bath (20 min) and then placed in a water bath (50 °C) until texture profile analysis. Three cooking replications were conducted. Three cooked unbroken rice grains were subjected to a two-cycle compression test using a TA.XT-Plus Texture Analyser equipped with a cylindrical probe (Ø = 35 mm, Stable Micro Systems Ltd., Surry, UK). The texture profile resulting from this two-compression test is composed of two sets of one positive and one negative curves, which can be divided into regions that represent downstrokes (increasing values) and upstrokes (decreasing values) (Additional file 2: Figure S2). *Hardness* (HRD, the peak of the first positive curve), *adhesiveness* (ADH, the area under the negative curve, representing the work required to pull the plunger from the sample on the base plate), *cohesiveness* (COH, the ratio of the area of the second positive curve to that of the first positive curve), and *springiness* (SPR, the ratio of the time elapsed from the upstroke to the peak in the second curve (T2) to the time elapsed from starting point to the peak of the first curve (T1), representing sample height recovery after the initial compression) [Lyon et al. 2000]. These parameters were measured at 90% strain and test speed at 0.5 mm s^− 1^. For each cooking replicate, three compression replicates were conducted.

### Protein content measurement

Crude protein content (PC) was measured using a modified protocol based on the automated colorimetric method (AACC Method 46–09) [AACC 2000]. A test portion of flour (50 mg) was digested in sulphuric acid (2 mL) with 1 g anhydrous potassium sulphate:selenium mixture (50:1, *w*/w) for 1 h at 370 °C. The sample was then cooled to room temperature and made up to volume (20 mL) with deionised water. It was kept overnight to allow for sedimentation. The liberated ammonium in the digest was then allowed to react with a solution containing sodium salicylate (0.94 M) and sodium nitroprusside (0.00026 M), and aqueous sodium hypochlorite (0.525%, also contained 30% Brij-35) while going through the glass transition lines in a San ++ Segmented Flow Analyser (SFA) system at 16 mL min^− 1^. Absorbance values of the ammonia-salicylate complex were determined at λ = 660 nm. The % Kjeldahl N values of the samples were determined based on the linear relationship between absorbance and analyte concentration, following the Beer-Lambert Law. This linear relationship was based on a standard curve developed using ammonium sulfate solutions with different concentrations. The crude PC was calculated by multiplying the Kjeldahl N value by 5.95 [Villareal et al. 1991].

### Statistics

Statistical analyses were carried out in R (version 3.3.2, released 2016). Ward’s cluster analysis was used to group the samples into three clusters based on 25 variables (AC, PC, RVA, advanced rheometry, and texture parameters). The raw dataset was used in developing a multinomial logistic regression (MLR) model to identify different clusters using Eq. 1:

1 $$ f\left( Xi,k\right)=\beta k\cdotp Xi, $$

where *f*(X_*i*_,*k*) is the score associated with the sample *i* assigned to cluster *k* (a non-binary categorical response variable), β_*k*_ is the vector of regression coefficients associated with cluster *k*, and X_*i*_ is the vector of explanatory variables describing sample *i*.

Tests of random forests (RF) were conducted with 500 trees and three variables randomly selected at each split. These random forests generated standardised scores that indicated the importance of each of the nine retained variables (determined by MLR) in classifying samples into the three clusters and also identified the most important variables per cluster. To rank the variables according to importance, the random forest algorithm determined the magnitude of increase in the prediction error (i.e., decrease in prediction accuracy) when the out-of-bag data were permuted (or excluded) for one variable while data for all other variables were held constant [Liaw and Wiener 2002; Louppe et al. 2013]. Hence, variables that had higher changes in magnitude of increases in prediction error were deemed more important than those variables that tend to have lower magnitudes.

### Sensory evaluation

Five samples from each cluster were selected for sensory evaluation using the texture profiling method [Lyon et al. 2000]. Milled grains from each sample were cooked using a 1:1 (*v*/v) ratio with water in rice cookers (0.6 L, Micromatic Model MRC-350). After the rice was cooked to completion, the rice was mixed, ensuring that the grains touching the sides and the bottom were undisturbed. Sub-samples were distributed into glass custard cups (pre-labelled with three-digit codes), sealed with a plastic lid, and then monadically presented for sensory evaluation to a previously trained set of panellists. Along with the sample, a tablespoon and a cup of drinking water were provided. To ensure that the samples were kept warm during the evaluation, the samples were kept in the rice cooker (at the “Warm” setting) and only placed in sample cups once the panellists requested for the samples. A rice breeding line, IR06N155 (harvested in the dry season of 2013 at IRRI’s Long-Term Continuous Cropping Experiment), was used as a standard. It was served to the panellists six times, randomly distributed in different rice tasting sessions.

The sensory panelists who participated in sensory evaluation were selected based on their availability and previous training in sensory descriptive profiling. The training phase included a battery of difference tests, sample and method familiarisation, and adjustment of the lexicon based on the panelists’ contexts [Champagne et al. 2010]. The rice samples used in the training phase were commercially available milled rice sold as Sinandomeng, Jasmine, and Long-grain rice.

The attributes tested during the rice tasting sessions represented the texture perceived at various stages of eating rice: from when the rice grains are first placed in the mouth up to after swallowing the rice. Panellists evaluate the intensity of each attribute on a 150-mm scale that has been adapted from established 15-point reference scales [Goodwin Jr. et al. 1996]. The R software was used for statistical analyses. Means and standard deviations were calculated per cluster.

## Results and discussion

This study characterized cooking quality properties of 211 diverse rice accessions based on 25 cooking quality variables including those routinely tested in grain quality evaluation programs (AC, GT, PC, and RVA parameters) along with specialized traits to capture viscoelastic properties measured by rheometry (Table 1) and textural properties measured by TPA. Previous publications indicate that many of these variables are correlated [e.g., Chung et al. 2011, Singh et al. 2006, Bao et al. 2006, Allahgholipour et al. 2006, Vandeputte et al. 2003, Tan and Corke 2002, Xie et al. 2011, Martin and Fitzgerald 2002] and may thus be treated as redundant variables. In this study, it was determined that 10 of the 25 variables were highly correlated (*r* > 0.75 or *r* < − 0.75, Table 2). Pasting temperature and temperature at the gel point were highly correlated with GT. Although peak viscosity (PV) was highly correlated with trough (TV) and final (FV) viscosities; FV was also highly correlated with lift-off (LO) and TV. In addition, G’_max_ was positively correlated with G” at G’_max_ and negatively correlated with temperature at G’_max_. Thus, six of these correlated attributes (PT, temperature at gel point, TV, FV, G” Temp at G’_max_) were removed as redundant from the subsequent analyses and 19 variables were retained, including GT, LO, G’_max_, and PV.

**Table 2:** Pearson correlation matrix

	GT	AC	PV	TV	BD	FV	SB	LO	PT	Pasting temp	HRD	ADH	COH	SPR
AC	− 0.05													
PV	0.08	−0.08												
TV	− 0.21**	0.05	0.88**											
BD	0.54**	−0.24**	0.48**	0.00										
FV	−0.10	0.10	0.88**	0.94**	0.11									
SB	−0.36**	0.34**	0.08	0.43**	−0.61**	0.55**								
LO	0.13	0.17*	0.59**	0.52**	0.29**	0.78**	0.58**							
PT	−0.37**	−0.10	0.48**	0.74**	−0.35**	0.56**	0.32**	0.03						
Pasting temp	0.82**	−0.01	0.01	−0.22**	0.43**	−0.17*	−0.37**	−0.01	−0.26**					
HRD	−0.12	0.20**	−0.01	0.03	−0.07	0.04	0.09	0.04	−0.05	− 0.14				
ADH	0.09	−0.31**	−0.03	−0.24**	0.38**	−0.29**	−0.56**	−0.29**	− 0.12	0.15*	0.22**			
COH	0.04	0.15*	0.21**	0.16*	0.14	0.20**	0.05	0.20**	−0.02	−0.01	0.36**	−0.08		
SPR	−0.06	0.33**	−0.03	−0.01	−0.04	0.02	0.09	0.06	−0.14	− 0.06	0.54**	−0.12	0.39**	
G’max	−0.34**	0.06	−0.17*	0.00	−0.35**	−0.01	0.28**	−0.03	0.11	−0.27**	−0.12	−0.15*	−0.08	0.02
G” at G’max	−0.11	0.13	−0.15*	−0.05	−0.22**	−0.06	0.13	−0.07	0.06	−0.05	−0.11	−0.10	−0.07	−0.03
Tan (δ) at G’max	0.20**	0.14	−0.03	− 0.07	0.07	−0.08	−0.12	−0.07	−0.05	0.25**	−0.01	0.06	−0.08	−0.07
Temp at gel point	0.75**	0.08	0.07	−0.13	0.37**	−0.07	−0.27**	0.05	−0.26**	0.65**	−0.04	−0.02	0.04	0.13
Temp at G’max	0.21**	−0.05	0.15*	0.09	0.14*	0.11	−0.04	0.10	0.01	0.14	0.09	−0.06	0.14*	0.01
G’trough	−0.26**	0.08	−0.10	0.07	−0.34**	0.05	0.29**	0.00	0.16*	−0.17*	−0.06	−0.14	0.05	0.04
PC	−0.12	−0.37**	−0.19*	0.01	−0.41**	−0.05	0.23**	−0.14*	0.17*	−0.14*	−0.01	−0.15*	−0.14	−0.14*
S1	−0.09	−0.01	−0.06	0.01	−0.14*	0.00	0.11	−0.02	0.07	−0.10	0.07	−0.03	0.12	0.03
S2	−0.12	0.03	−0.06	0.04	−0.20**	0.04	0.18*	0.02	0.06	−0.09	0.01	−0.12	0.05	−0.04
S3	0.05	0.02	−0.03	0.02	−0.08	0.03	0.11	0.05	−0.02	0.03	−0.04	−0.19*	0.05	−0.02
S4	−0.06	0.01	0.01	0.07	−0.11	0.04	0.07	−0.03	0.13	−0.09	0.00	−0.02	0.11	−0.04
	G’max	G” at G’max	Tan (δ) at G’max	Temp at gel point	Temp at G’max	G’trough	PC	S1	S2	S3				
AC														
PV														
TV														
BD														
FV														
SB														
LO														
PT														
Pasting temp														
HRD														
ADH														
COH														
SPR														
G’max														
G” at G’max	0.78**													
Tan (δ) at G’max	−0.12	0.50**												
Temp at gel point	−0.43**	−0.26**	0.12											
Temp at G’max	−0.79**	−0.73**	−0.15*	0.27**										
G’trough	0.54**	0.44**	−0.04	−0.29**	−0.30**									
PC	0.16*	0.01	−0.19*	−0.17*	0.05	0.20**								
S1	0.39**	0.31**	−0.01	−0.16*	−0.28**	0.18*	0.04							
S2	0.27**	0.19*	−0.03	−0.16*	−0.06	0.13	0.10	0.33**						
S3	0.33**	0.34**	0.08	−0.06	−0.20**	0.25**	0.06	0.39**	0.34**					
S4	0.02	−0.04	−0.10	−0.16*	0.16*	0.11	0.01	0.34**	0.29**	0.49**				

*p < 0.1, ** *p* < 0.05, *** *p* < 0.01

**Fig. 1:**
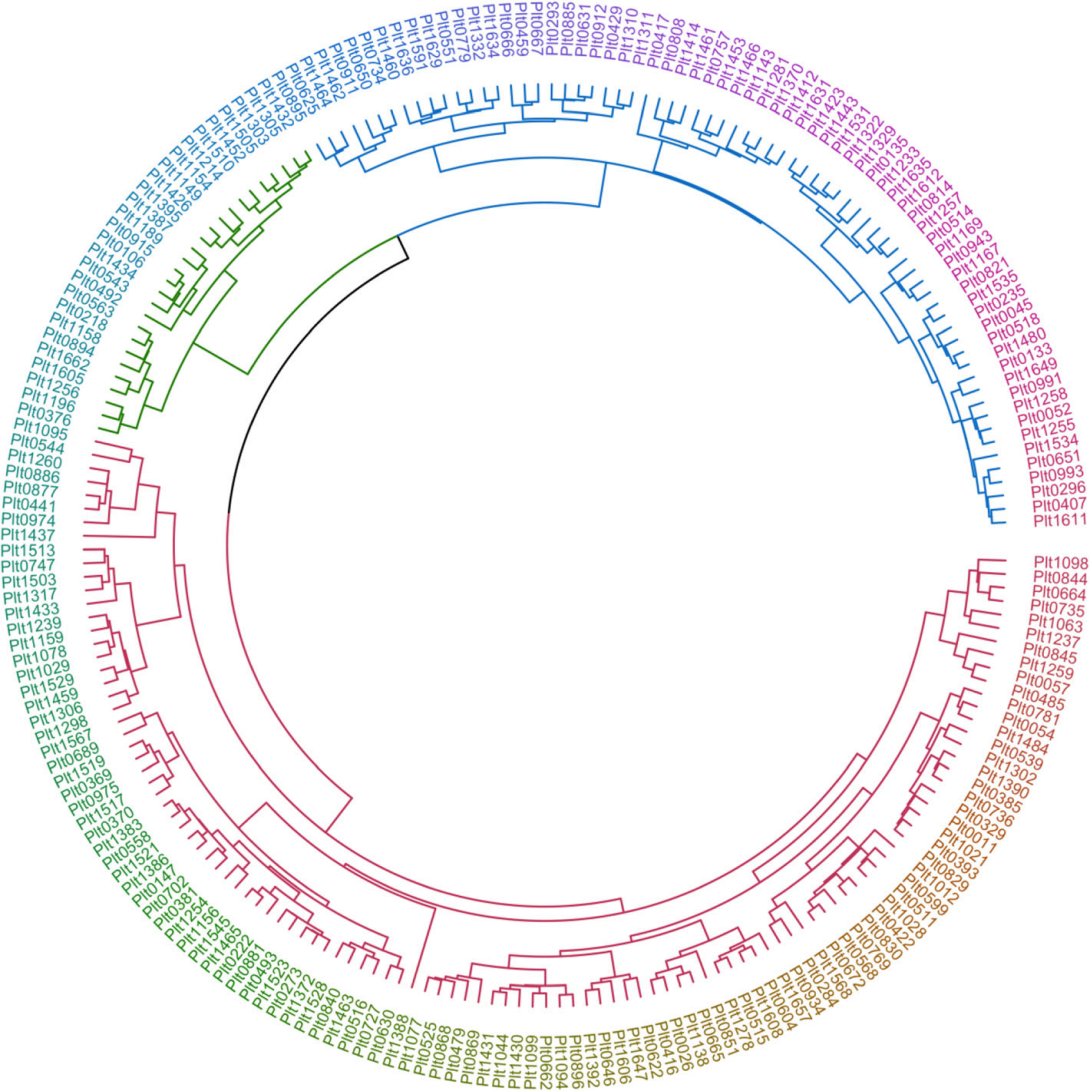
Ward’s cluster analysis indicates that the samples (*n* = 211) grouped into three clusters based on 19 grain quality attributes: n_1_ = 114 (red), n_2_ = 70 (blue), n_3_ = 27 (green)

By employing Ward’s cluster analysis to 19 variables (Table 2), three distinct clusters were identified (Fig. 1). Among them, cluster 1 was the biggest, with 114 samples, followed by cluster 2 with 70 samples, and by cluster 3 with 27 samples.

**Fig. 2:**
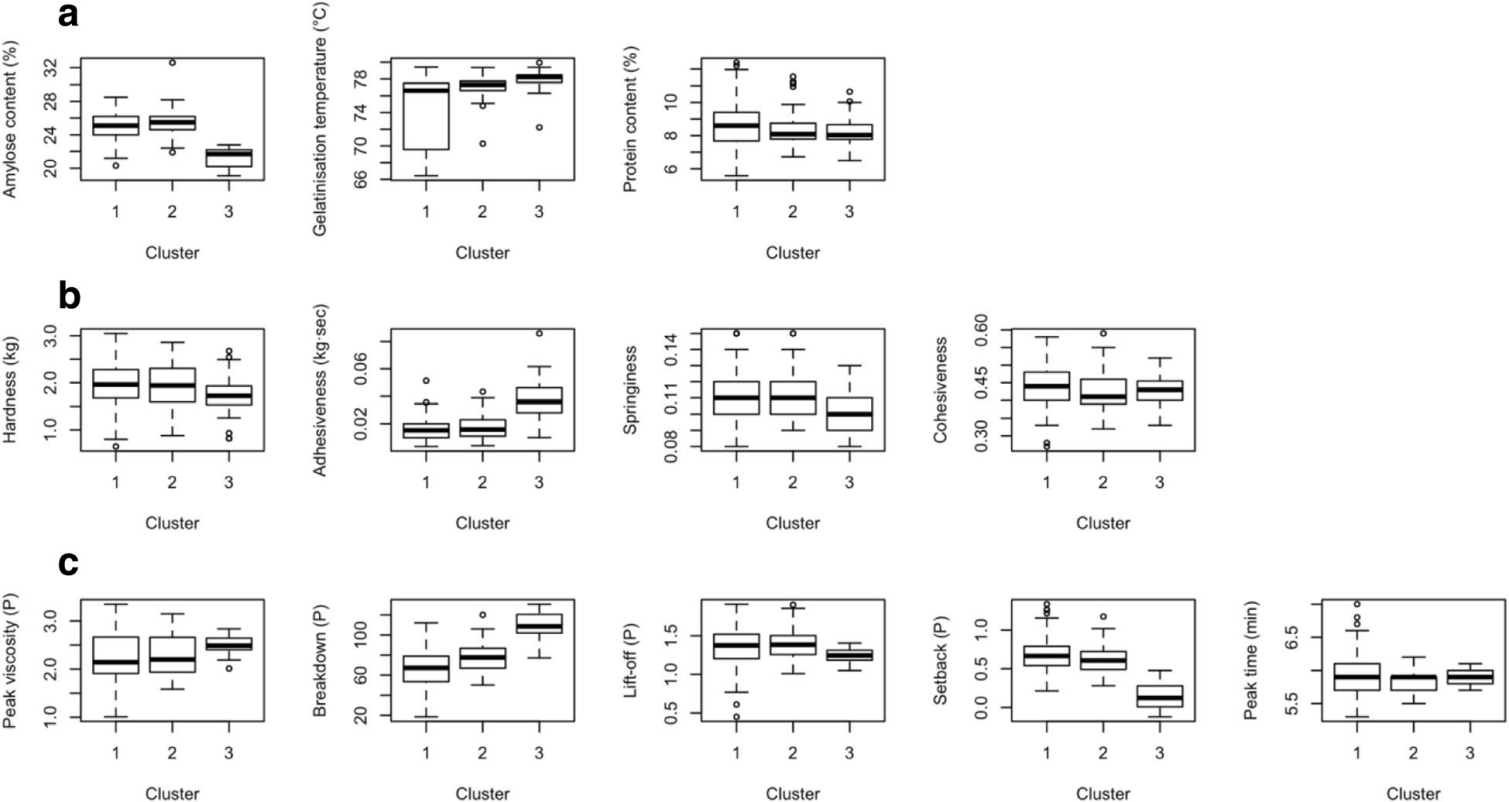
Boxplots of the three clusters of rice samples for (**a**) GT, AC, and PC; (**b**) TPA parameters: HRD, ADH, COH, SPR; (**c**) RVA parameters PV, BD, LO, SB, and Peak time; and

Clusters 1 and 2 have high AC; on the other hand, samples in cluster 3 could clearly be classified as intermediate AC (Table 3, Fig. 2a). Due to the similarities in ranges of AC between clusters 1 and 2, it is important to distinguish these two clusters based on visco-elastic parameters, through advance rheometry, and on textural attributes. In the past, rice samples with similar ACs have been reported to have distinguishable textural properties [e.g., Champagne et al. 2010; Champagne et al. 1999]; hence, it is important to explore other indicators of cooking behaviors’ and organoleptic properties in order to fine tune how rice varieties within the high AC classes are classified.

**Table 3:** Means of 19 grain quality indicators of rice samples, by cluster. Standard deviations are indicated in parentheses

Variable	Cluster 1 (*n* = 114)		Cluster 2 (*n* = 70)		Cluster 3 (*n* = 27)	
GT (°C)	74.81	(3.98)	77.10	(1.22)	77.94	(1.38)
AC (%)	25.07	(1.44)	25.51	(1.49)	21.22	(1.24)
PV (P)	2.23	(0.52)	2.28	(0.43)	2.49	(0.18)
BD (P)	0.67	(0.20)	0.78	(0.14)	1.10	(0.14)
SB (P)	0.69	(0.21)	0.61	(0.18)	0.15	(0.18)
LO (P)	1.36	(0.25)	1.39	(0.19)	1.24	(0.09)
PT (min)	5.96	(0.30)	5.84	(0.18)	5.89	(0.13)
HRD (kg)	1.96	(0.50)	1.94	(0.50)	1.73	(0.47)
ADH (kg·sec)^a^	0.02	(0.01)	0.02	(0.01)	0.04	(0.02)
COH	0.44	(0.06)	0.42	(0.05)	0.43	(0.04)
SPR	0.11	(0.02)	0.11	(0.01)	0.10	(0.01)
G’_max_ (kPa)	40.64	(9.62)	28.34	(7.20)	29.83	(7.26)
tan (δ) at G’_max_	0.11	(0.02)	0.12	(0.04)	0.11	(0.02)
G’_trough_ (kPa)	15.53	(4.31)	11.92	(2.33)	11.66	(1.83)
S1 (kPa/min)	7.45	(2.66)	4.66	(1.88)	5.93	(2.49)
S2 (kPa/min)^a^	1.66	(0.58)	1.27	(0.33)	1.29	(0.22)
S3 (kPa/min)	1.77	(0.70)	1.19	(0.58)	1.38	(0.63)
S4 (kPa/min)^a^	1.53	(0.81)	1.09	(0.63)	1.24	(0.71)
PC (%)	8.66	(1.26)	8.34	(1.02)	8.30	(1.00)

^a^Absolute values are indicated here because for these parameters, the negative (−) sign only indicates direction (i.e., up or down) rather than magnitude less than zero

Gelatinisation temperature is (GT) often regarded as one of the most important factors affecting cooked rice quality and it is often viewed in combination with AC because an increase in AC reportedly leads to elevated GT [Juliano et al. 2009; Yang et al. 2014]. In this study, cluster 2 (high AC) and cluster 3 (intermediate AC) were clearly classified as high GT; on the other hand, cluster 1 (high AC) has been classified as low GT (Fig. 2a, Table 3). This indicates that AC alone cannot contribute to increase in GT (Table 2). GT in addition, might potentially be influenced by medium chain length contribution of amylopectin (Miura et al. 2018). In this context, starch structure would play an important role to fine-tune the cooking quality ideotypes of the samples.

Protein content (PC) has been reported to affect cooked rice stickiness [Champagne et al. 2009] and surface hardness [Okadome 2005]. In this study, however, the averages of PC for the three clusters ranged from 8.30 to 8.66% (Table 3), suggesting that PC is probably not a discriminatory factor for clustering these samples. While clusters 2 and 3 had similar ranges of PC, the cluster 1 appeared to have the widest range in PC (Fig. 2a). These results agree with a previously published report that PC was not an attribute that can differentiate cooking quality classes within rice collections [Bett-Garber et al. 2001].

The TPA provided measurements for HRD, ADH, COH, and SPR. The three clusters had similar values for HRD (Table 3), with ranges also observed in waxy rice [Boualaphanh et al. 2011]. This indicates further that AC did not solely affect HRD for the samples in this study, as these two parameters are weakly correlated (Table 2). However, due to the similarities in values, HRD potentially could not define the three quality clusters. Diversity lines differ for ADH ranged from 0.02 to 0.04 kg·sec, COH ranged from 0.42 to 0.44, and SPR from 0.10 to 0.11 (Table 3). The box plots indicated that the ranges of the textural attributes represented in clusters 1 and 2 overlapped such that it was difficult to separate the two clusters from each other (Fig. 2b).

Pasting parameters are additional indicators of organoleptic quality, with parameters extensively studied particularly with their relationships with AC, PC, and mechanical texture attributes [reviewed in Champagne et al. 1999, Okadome et al. 2005, Yoenyongbuddhagal and Noomhorm 2002]. Results indicated that the clusters 1 and 2 exhibited similar averages for the different pasting parameters (Table 3); however, the averages for BD and SB clearly distinguish cluster 3 from the other two clusters (Table 3). Cluster 3 had the lowest setback among the three clusters (Table 3). This agrees with previous reports that indicate that setback and AC are correlated [e.g., Allahgholipour et al. 2006; Tan and Corke 2002; Chen et al. 2008]. Cluster 3 also has the highest BD (Table 3), indicating that the samples in this cluster are most resilient to continuous agitation stress.

**Fig. 3:**
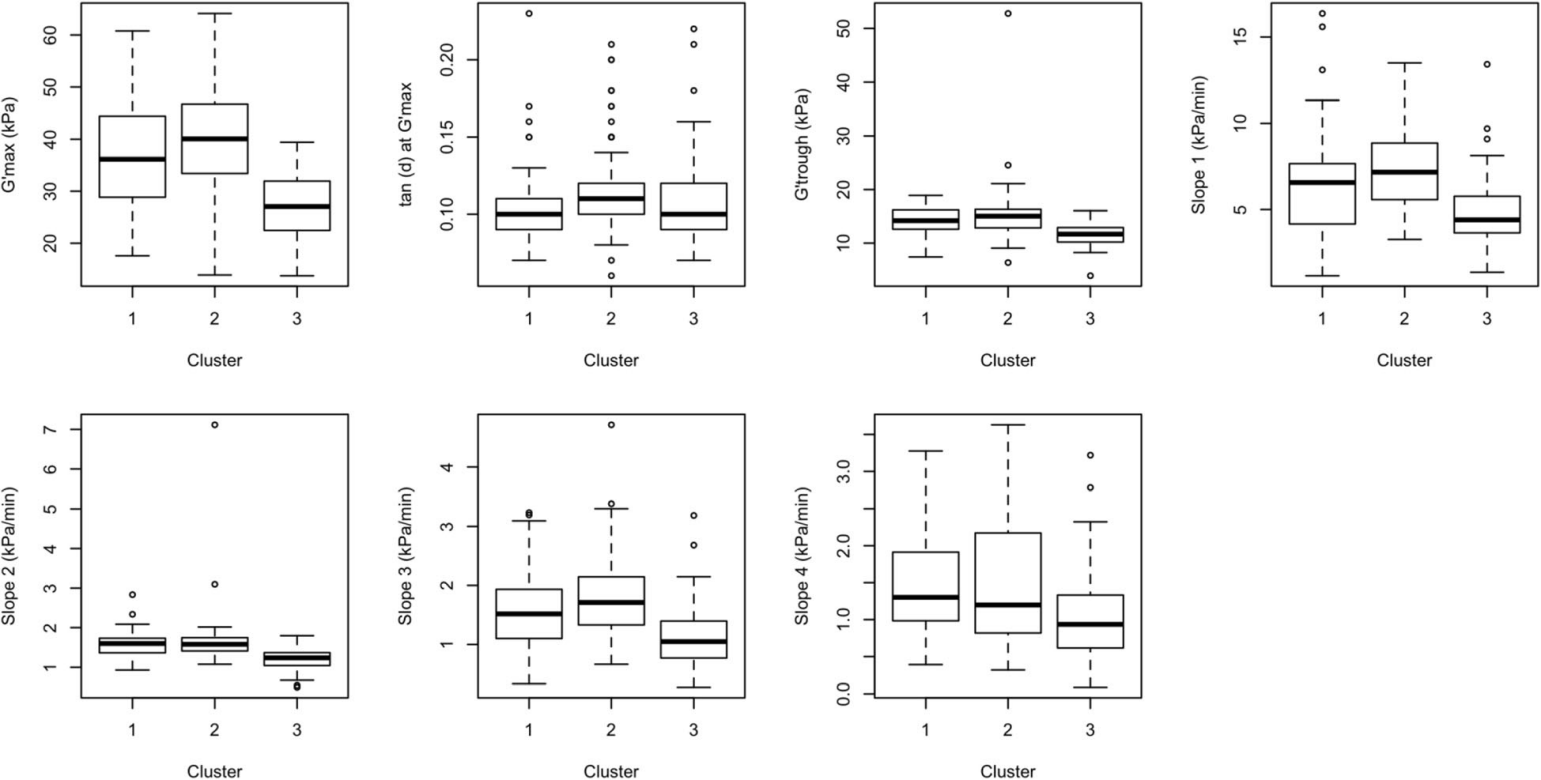
Boxplots of the three clusters of rice samples for rheometry parameters: G’_max_, tan (δ) at G’_max_, G’_trough_, Slope 1 (G’), Slope 2 (G’), Slope 3 (G”), Slope 4 (G”)

Although most of the traditional grain quality parameters (AC, PC, GT and viscosity profiles of RVA) could not distinguish high-AC accessions represented within clusters 1 and 2, rheometry parameters have shown a nice range of differentiation between cluster 1 and 2 (Table 3 and Fig. 3). Cluster 1 represented lines had the highest averages for G’_max_ and G’_trough_ while the other two clusters had similar values. Likewise, cluster 1 could clearly be distinguished from cluster 2 and cluster 3 based on S1, S2, S3, and S4 derived from the rheometer curves (Table 3 and Fig. 3).

In breeding programs, rice cooking quality classification is typically based on AC, GC, and GT considered individually [e.g., Juliano et al. 2009; Bett-Garber et al. 2001]. Although influence of AC on cooking quality is helpful when considering diverse rice collections containing both japonica and indica rice, the classical means of quality classification will probably not work when dealing with rice varieties within the same AC class in an indica germplasm collection. This study, therefore, used MLR (Eq. 1) in categorising the samples within the high-AC groups by including multiple variables in a classification model for cooking quality ideotypes. The “Forward Selection (Akaike Information Criterion, AIC)” allows stepwise selection to exclude the multi-collinear variables from the model. Through this step, 10 out of 19 variables were retained in the model, most of which were significant (*p* <  0.05) in the likelihood ratio test (Table 4). The 10 attributes which contributed significantly to the model included AC and GT from routine grain quality parameters; BD from RVA; G’_max,_ tan (δ), G’_trough_, S1, S2, and S3 from rheometry; and COH from TPA. The model has high classification accuracy (93.84%, Table 5) with sufficient explanatory power, as indicated by the change in –2Loglikelihood in the final model (χ^2^ = 339.66, df = 18, *p* <  0.01, Table 5). Also, the pseudo-R^2^ values indicate high levels of fit to differentiate clusters represented by cooking quality groups (Table 5).

**Table 4:** Likelihood ratio (LR) test

Variable	LR Chisq	Pr (> Chisq)
AC	58.95	< 0.01***
G’_trough_	9.25	0.01**
BD	7.80	0.02*
S1	20.87	< 0.01***
tan (δ) at G’_max_	24.06	< 0.01***
S3	15.35	< 0.01***
GT	13.30	< 0.01**
COH	13.91	< 0.01***
G’_max_	9.95	0.01**
S2	4.14	0.13

* *p* < 0.05, ** p < 0.01

Degrees of Freedom = 2

**Table 5:** Summary of multinomial logistic regression for variables characterising the different rice quality clusters. Cluster 1 is not shown in Table 5 because it is the reference cluster. Table 5 indicates the multinomial log-odds that samples represented in Cluster 2 or in Cluster 3, were compared to reference cluster 1 to calculate every unit increase or decrease in the different grain quality attributes included in the multinomial logistic regression model

Grain quality attribute	Estimate			
	Cluster 2		Cluster 3	
Intercept	−25.51	(15.27)	3.61	(0.08)***
AC (%)	0.08	(0.30)	−9.17	(2.94)***
G’_trough_	−0.33	(0.19)*	−1.48	(0.93)
BD	0.06	(0.03)**	0.72	(3.55)
S1	−0.88	(0.22)***	−1.37	(3.20)
tan (δ) at G’_max_	0.55	(0.14)***	0.21	(11.71)
S3	−2.84	(0.84)***	−0.12	(0.66)
GT	0.54	(0.18)***	2.03	(6.97)
COH	−0.24	(0.08)***	0.01	(11.97)
G’_max_	−0.15	(0.06)***	0.22	(8.68)
N	70		27	

Note: Total N = 211; AIC = 106.20; Overall classification accuracy: 93.84%

Reference category for the regression model is cluster 1 (n = 114)

Standard errors of the estimates are indicated in parentheses

* *p* < 0.1, ** *p* < 0.05, *** *p* < 0.01.

Goodness-of-fit statistics: Residual Deviance = 66.20; Degrees of freedom = 18

–2Log-likelihood: The intercept-only model: 405.87; The final model: 66.20; χ^2^ = 339.66; *p* < 0.01

Pseudo-R^2^: McFadden = 0.84; Cragg & Uhler = 0.94; Cox & Snell = 0.80

**Fig. 4:**
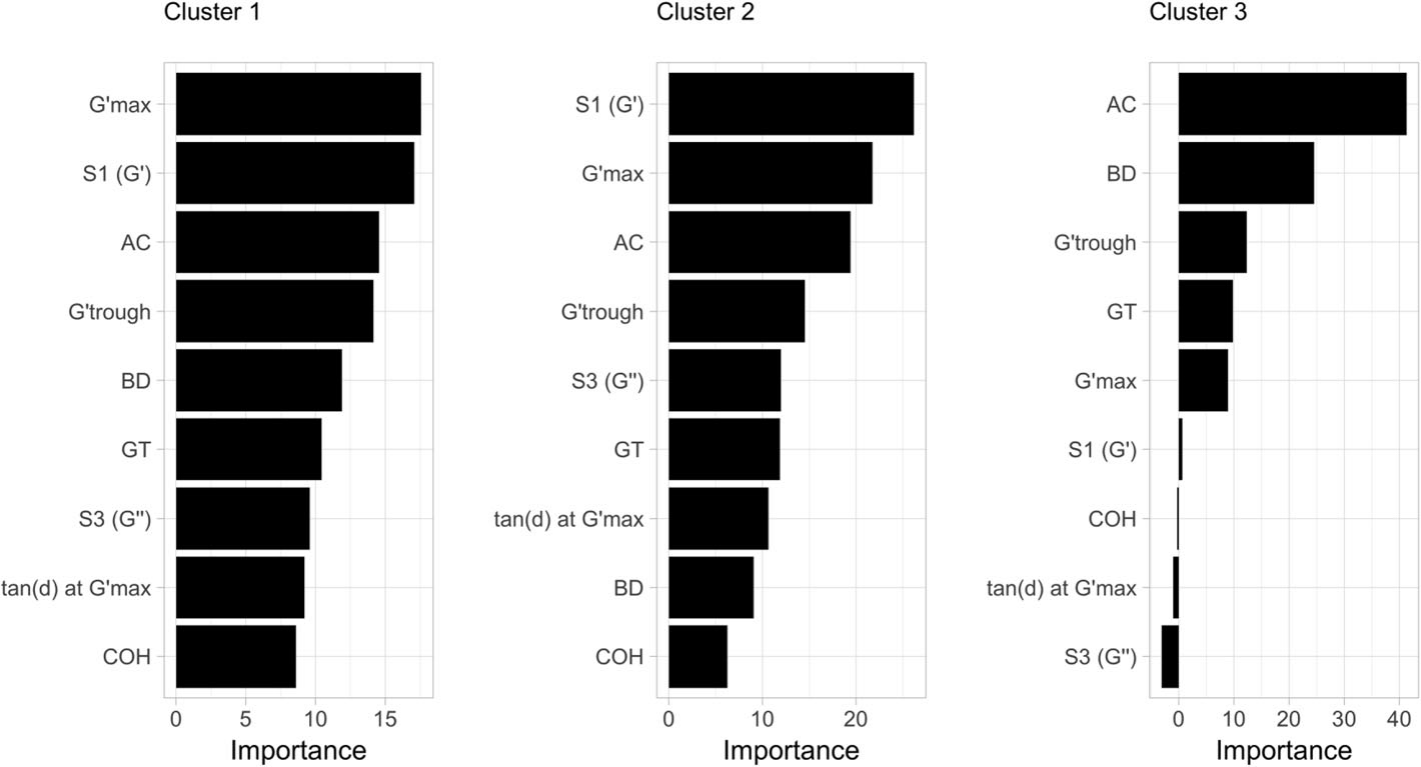
Importance of the nine grain quality variables included in the final MLR model in each cluster, as calculated using Random Forests

The magnitudes of the coefficients (i.e., multinomial log-odds) differed across clusters (Table 5); however, these do not indicate the importance of these variables in explaining the model. The relative importance of these variables was determined via random forest. Results show that AC was the most important variable in defining Cluster 3 (Fig. 4); this is expected because this cluster has the lowest AC values (Fig. 2a and Table 3). On the other hand, the two most important variables differentiating clusters 1 and 2 were rheometry parameters, S1 and G’_max_. Amylose content ranked third in importance for both clusters, perhaps because AC can differentiate these two clusters from Cluster 3. The MLR model (Table 5) indicates that for every unit increase in BD, tan (δ) at G’_max_, and GT, the multinomial log-odds distinguished accessions represented in cluster 1 from cluster 2. Meanwhile, for every unit increase in G’_trough_, S1, S3, COH, and G’_max_, the multinomial log-odds that the sample belonged to cluster 2 rather than to cluster 1 decreased.

**Fig. 5:**
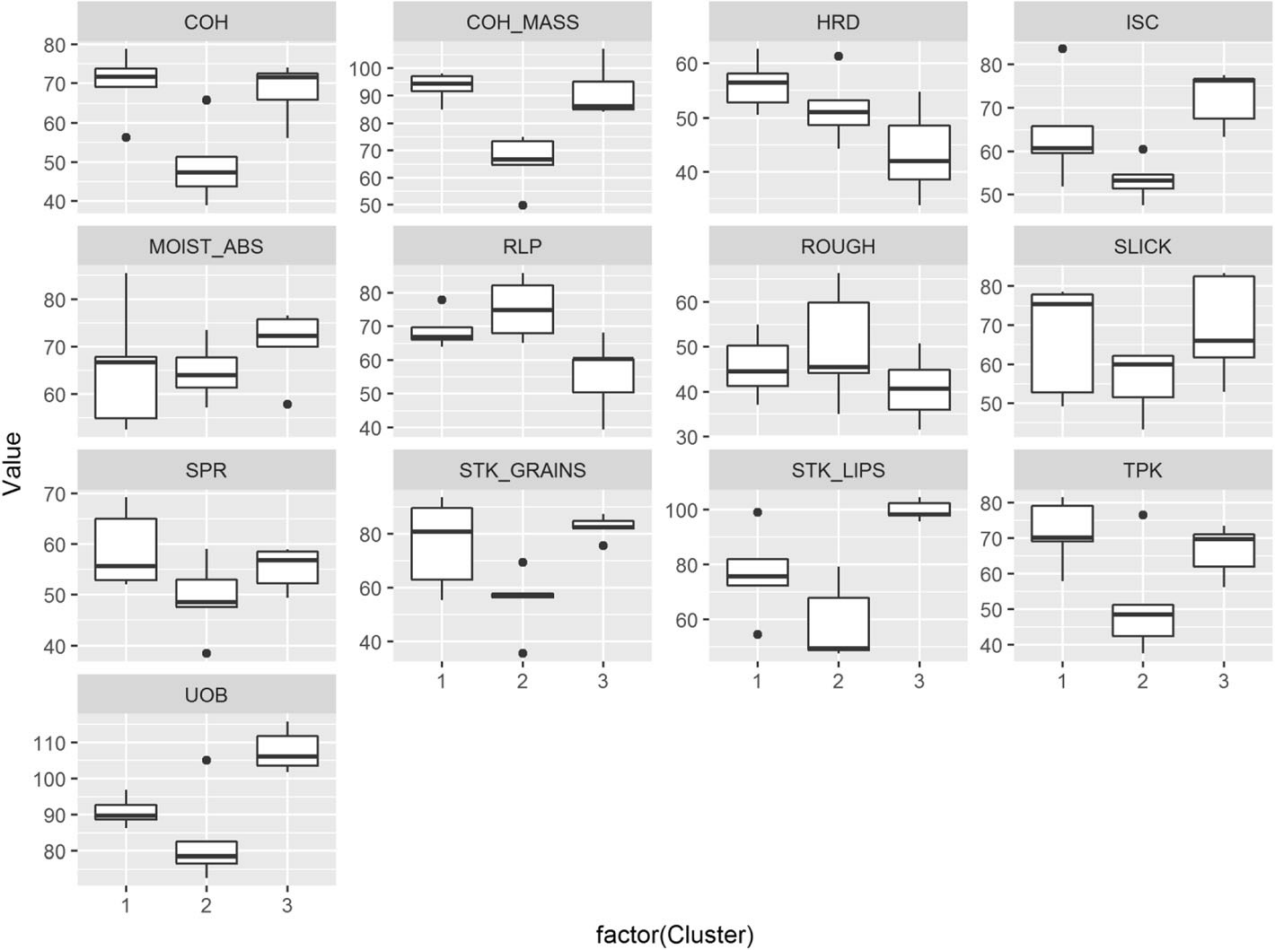
Box plots comparing the three clusters based on 13 texture attributes evaluated by sensory panelists based on a 150-mm scale. The sensory attributes [13] evaluated were: cohesiveness (COH), cohesiveness of mass (COH_MASS), hardness (HRD), initial starchy coating (ISC), moisture absorption (MOIST_ABS), residual loose particles (RLP), roughness (ROUGH), slickness (SLICK), springiness (SPR), stickiness between grains (STK_GRAINS), stickiness to the lips (STK_LIPS), toothpack (TPK), uniformity of bite (UOB)

The capacity to differentiate between rice varieties within the same AC class through instrumental means becomes truly important if the differences can be detected by rice consumers. Hence, five samples from each cluster were subjected to descriptive profiling for texture by a trained sensory panel (Table 6, Additional file 3: Table S1). The sensory profiles generated for textural attributes were compared (Fig. 5, Table 7). It was notable that clusters 1 and 2 had similar ranges of AC but had distinguishable sensory attributes. There was a remarkable difference between clusters 1 and 2 in terms of stickiness (both to the lips and between grains), cohesiveness, cohesiveness of mass, toothpack, and uniformity of bite. Furthermore, accessions representing cluster 1 were perceived to be slightly harder and springier than accessions from cluster 2, although both have high AC ranges. These results suggest that lines represented in clusters 1 and 2, distinguished initially based on rheometry and mechanical texture properties, could be differentiated by humans (Table 7). This further suggests that there may be relationships among force-related textural attributes perceived by people, rheological properties, and those attributes measured by texture profile analyses; and these attributes may distinguish lines with high-AC content. The differences in sensory profiles between clusters 1 and 3 appear to be related to moisture absorption, residual loose particles, and initial starchy coating (Fig. 5). Results also indicate that, despite the difference in AC class, accessions representing cluster 3 were similar to samples in cluster 1 in terms of cohesiveness, cohesiveness of mass, roughness, slickness, stickiness between grains, and toothpack. Attributes such as cohesiveness, cohesiveness of mass, toothpack, and uniformity of bite have not been explored as deeply as the force-related textural properties.

**Table 6:** Samples that underwent descriptive sensory profiling from the three clusters and their values for AC, GT, and PC. IDs linked to accession names have been described in Additional file 3: Table S1

Cluster Number	Sample	AC (%)	GT (°C)	PC (%)
1	GQ 00403 Plt 0057	24.5	68.44	8.27
	GQ 01652 Plt 0222	24.6	77.92	8.03
	GQ 01524 Plt 0369	27.4	76.88	6.55
	GQ 01633 Plt 0370	27.2	76.44	6.84
	GQ 01613 Plt 0493	26.3	77.81	7.74
2	GQ 00261 Plt 0885	26.8	76.60	9.40
	GQ 00324 Plt 0993	25.2	77.75	8.63
	GQ 00089 Plt 1143	25.5	76.89	8.33
	GQ 00401 Plt 1167	23.9	77.17	11.13
	GQ 00131 Plt 1414	25.0	78.45	7.38
3	GQ 01527 Plt 0106	22.7	78.24	7.74
	GQ 01523 Plt 0218	20.5	77.58	7.91
	GQ 01696 Plt 0376	19.4	78.65	8.63
	GQ 01659 Plt 0492	19.9	78.43	8.33
	GQ 01691 Plt 0543	22.7	79.94	6.49

**Table 7:** Comparison of intensities of sensory attributes based on descriptive test conducted through panel evaluation

Sensory attribute	Cluster		
	1	2	3
Initial starchy coating	64.3±11.9	53.4±4.8	72.3±6.4
Slickness	66.8±14.5	55.8±8.3	69.3±13.3
Roughness	45.6±7.1	50.2±12.7	40.8±7.5
Stickiness to the lips	76.7±16.1	58.6±14.2	99.6±3.5
Stickiness between grains	76.6±16.6	55.4±12.2	82.5±4.4
Springiness	59.0±7.7	49.3±7.6	55.2±4.2
Cohesiveness	69.9±8.4	49.4±10.2	68.0±7.3
Hardness	56.1±4.7	51.7±6.3	43.5±8.3
Uniformity of bite	93.3±5.2	65.9±10.0	91.6±9.7
Cohesiveness of mass	90.8±4.1	83.1±12.8	107.8±5.8
Moisture absorption	65.5±13.1	64.7±6.3	70.5±7.6
Residual loose particles	68.9±5.4	75.1±8.9	55.7±11.1
Toothpack	71.5±9.4	51.2±15.1	66.4±7.2

## Conclusions

To predict cooking quality ideotypes of indica rice with intermediate-to-high AC, we used 25 variables covering routine cooking quality predictors, RVA, rheometry, and instrumental texture profiling. Results showed that these intermediate- and high-AC samples could be classified into two distinct clusters using 19 variables. Clusters 1 and 2 both contained samples with high-AC while cluster 3 had samples with intermediate AC. This is the first study in which MLR was used to further differentiate high-AC samples using rheometry, TPA, and RVA parameters simultaneously. The differences in sensory profiles between the two clusters validate the use of rheometric properties as proxy metrics to distinguish cooking and eating quality within high-AC ranges. This study calls for a deeper look into variables extracted from rheometry and descriptive sensory evaluation, as these could enhance our capacity to classify rice into quality classes that match consumer preferences. A deeper understanding about these attributes will be important as breeding strategies become increasingly reliant on product profiles. The capacity to measure these attributes in an efficient and quantitative manner can also help set standards that can be used for developing policy and trade recommendations to capture the cooking and eating quality properties of rice in varietal development programs. These rice quality recommendations will be handy as rice-growing countries continue to strive to supply domestic and export needs.

## Additional files

Additional file 1: **Table S4.** Phenotype data of grain quality cooking and eating quality parameters, rapid viscosity analyzer, advance rheometry and texture attributes of core collection panel. (XLSX 66 kb)

Additional file 2: **Figure S1.** Storage (G’) and loss moduli (G”) curves of GQ00043-PLT1298 (1:2 w/vflour: water ratio) obtained during heating and cooling steps in a rheometer. The parameters defined in therheometry profiles are described in Table 1. **Figure S2.** A typical two-compression TPA curve obtained from a texture analyzer. Hardness(kg) is peak force of the first compression (H1). Adhesiveness is the negative force area of the first bite.Cohesiveness is the ratio of the positive force area during the second compression portion to that during thefirst compression (A2/A1). Springiness is defined as the ratio of T2 to T1, where T1 is total distance travelledby the probe on downstroke and T2 is distance traveled on downstroke by the probe from point of samplecontact to end of downstroke (T2/T1). (ZIP 214 kb)

Additional file 3: **Table S1.** Designations of samples used for sensory evaluation, selected from the three clusters. **Table S2.** Data used for multivariate analyses for the samples selected for sensory evaluation. **Table S3.** Sensory evaluation scores^1^ for the fifteen rice accessions from the three clusters. (DOCX 30 kb)

## Abbreviations

AC: Amylose content
ADH: Adhesiveness
COH: Cohesiveness
DSC: Differential scanning calorimetry
FV: Final viscosity
GT: Gelatinisation temperature
HRD: Hardness
LO: Lift-off
MLR: Multinomial Logistic Regression
PC: Protein content
PV: Peak viscosity
RVA: Rapid Visco-Analysis
SB: Setback
SPR: Springiness
TPA: Texture profile analysis
TV: Trough viscosity
