# Supplementary figures and images for: Multivariate-based classification of predicting cooking quality ideotypes in rice (Oryza sativa L.) indica germplasm
Source: Rice (N Y). 2018 Oct 10;11:56. doi: 10.1186/s12284-018-0245-y (PMC6179975; doi:10.1186/s12284-018-0245-y)

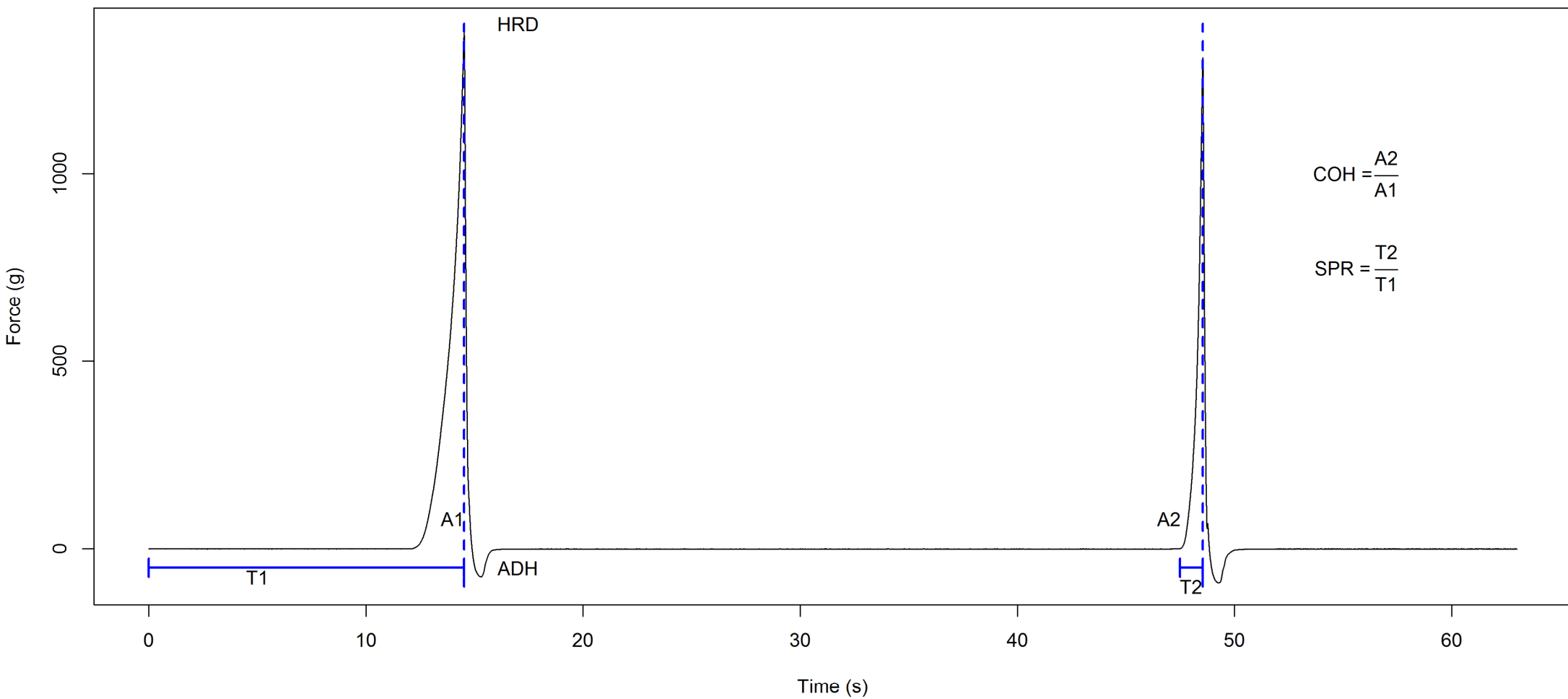

Supplement: Supplementary file 2 — Figure S1. Storage (G’) and loss moduli (G”) curves of GQ00043-PLT1298 (1:2 w/vflour: water ratio) obtained during heating and cooling steps in a rheometer. The parameters defined in therheometry profiles are described in Table 1. Figure S2. A typical two-compression TPA curve obtained from a texture analyzer. Hardness(kg) is peak force of the first compression (H1). Adhesiveness is the negative force area of the first bite.Cohesiveness is the ratio of the positive force area during the second compression portion to that during thefirst compression (A2/A1). Springiness is defined as the ratio of T2 to T1, where T1 is total distance travelledby the probe on downstroke and T2 is distance traveled on downstroke by the probe from point of samplecontact to end of downstroke (T2/T1). (ZIP 214 kb) [file 12284_2018_245_MOESM2_ESM.zip › Figure S2.pdf]

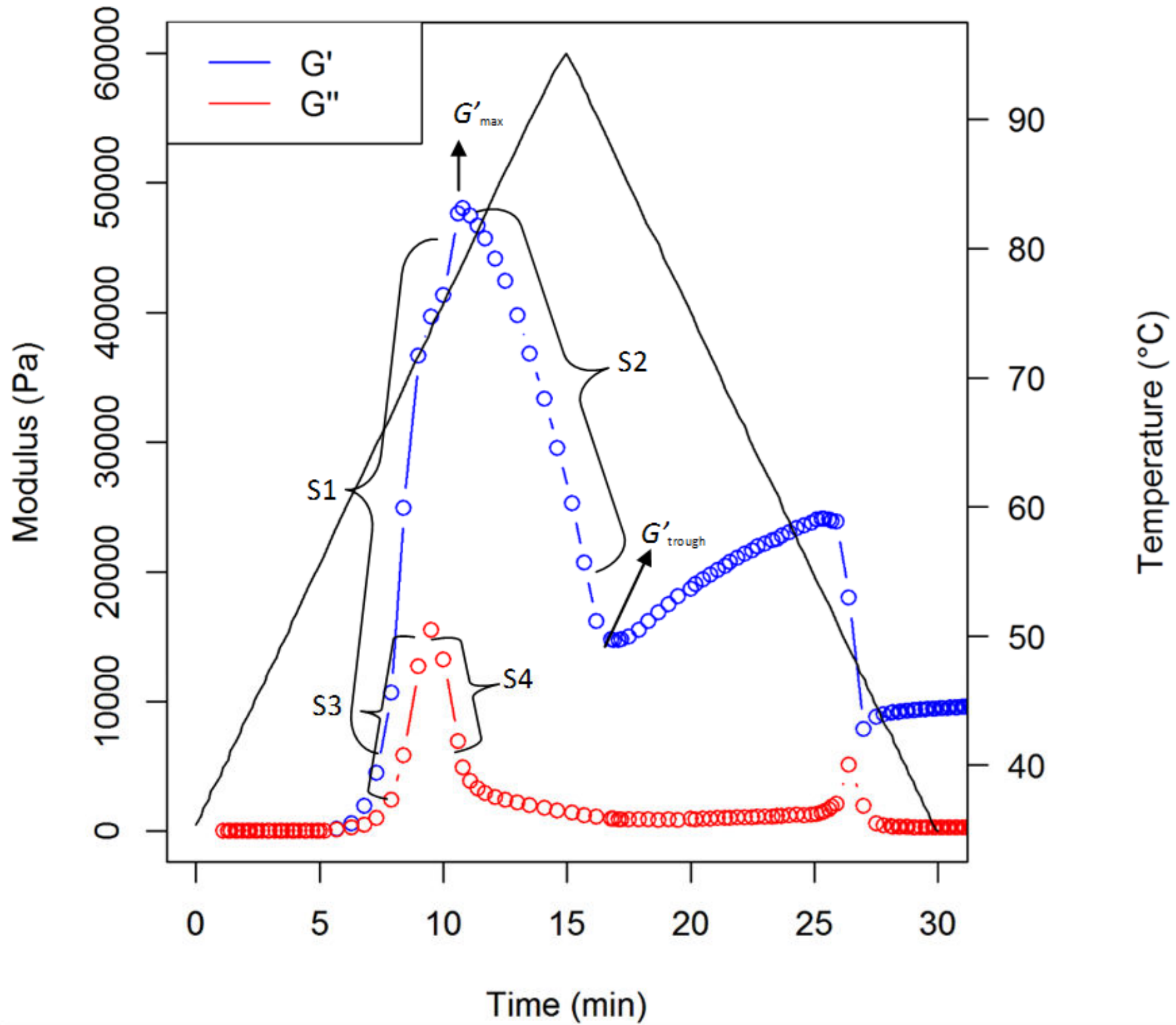

Supplement: Supplementary file 2 — Figure S1. Storage (G’) and loss moduli (G”) curves of GQ00043-PLT1298 (1:2 w/vflour: water ratio) obtained during heating and cooling steps in a rheometer. The parameters defined in therheometry profiles are described in Table 1. Figure S2. A typical two-compression TPA curve obtained from a texture analyzer. Hardness(kg) is peak force of the first compression (H1). Adhesiveness is the negative force area of the first bite.Cohesiveness is the ratio of the positive force area during the second compression portion to that during thefirst compression (A2/A1). Springiness is defined as the ratio of T2 to T1, where T1 is total distance travelledby the probe on downstroke and T2 is distance traveled on downstroke by the probe from point of samplecontact to end of downstroke (T2/T1). (ZIP 214 kb) [file 12284_2018_245_MOESM2_ESM.zip › Figure S1.pdf]
