# Supplementary material for: Multivariate-based classification of predicting cooking quality ideotypes in rice (Oryza sativa L.) indica germplasm
Source: Rice (N Y). 2018 Oct 10;11:56. doi: 10.1186/s12284-018-0245-y (PMC6179975; doi:10.1186/s12284-018-0245-y)
Supplement: Supplementary file 3 — Table S1. Designations of samples used for sensory evaluation, selected from the three clusters. Table S2. Data used for multivariate analyses for the samples selected for sensory evaluation. Table S3. Sensory evaluation scores1 for the fifteen rice accessions from the three clusters. (DOCX 30 kb) [file 12284_2018_245_MOESM3_ESM.docx]

Table S1. Designations of samples used for sensory evaluation, selected from the three clusters.

| Cluster | Sample | Designation |
| --- | --- | --- |
| 1 | GQ 00403 Plt 0057 | ARC 15027::IRGC 43043-1 |
|  | GQ 01652 Plt 0222 | WAB 56-125 |
|  | GQ 01524 Plt 0369 | FEDEARROZ 50 |
|  | GQ 01633 Plt 0370 | IRRI 165 |
|  | GQ 01613 Plt 0493 | IRRI 119 |
|  |  |  |
| 2 | GQ 00261 Plt 0885 | IR 50::C 1 |
|  | GQ 00324 Plt 0993 | IR 77390-6-1 |
|  | GQ 00089 Plt 1143 | DA 5::IRGC 5855-1 |
|  | GQ 00401 Plt 1167 | ARC 13856::IRGC 41266-1 |
|  | GQ 00131 Plt 1414 | LOKU SAMBA:IRGC 31462-1 |
|  |  |  |
| 3 | GQ 01527 Plt 0106 | INIA TACUARI |
|  | GQ 01523 Plt 0218 | CYPRESS |
|  | GQ 01696 Plt 0376 | PSBRC 18 SUB 1 |
|  | GQ 01659 Plt 0492 | CIHERANG SUB 1 |
|  | GQ 01691 Plt 0543 | IRRI 146 |

Table S2. Data used for multivariate analyses for the samples selected for sensory evaluation.

| Cluster | Sample | GT (ºC) | AC (%) | PV (P) | TV (P) | BD (P) | FV (P) | SB (P) | LO (P) | PT (min) | PASTEMP (ºC) | HRD (kg) | ADH (kg sec) | COH |
| --- | --- | --- | --- | --- | --- | --- | --- | --- | --- | --- | --- | --- | --- | --- |
| 1 | GQ 00403 Plt 0057 | 68.44 | 24.5 | 126.92 | 77.5 | 49.42 | 168 | 41.08 | 90.5 | 5.7 | 70.45 | 1806.45 | 7.6 | 0.42 |
|  | GQ 01652 Plt 0222 | 77.92 | 24.6 | 281.08 | 176.25 | 104.83 | 344.25 | 63.17 | 168 | 5.8 | 79.15 | 2646.12 | 17.38 | 0.51 |
|  | GQ 01524 Plt 0369 | 76.88 | 27.4 | 225.67 | 134.58 | 91.08 | 281.08 | 55.42 | 146.5 | 5.7 | 79.15 | 1967.39 | 18.76 | 0.45 |
|  | GQ 01633 Plt 0370 | 76.44 | 27.2 | 240.58 | 166.67 | 73.92 | 289.17 | 48.58 | 122.5 | 6.1 | 79.8 | 2304.85 | 31.98 | 0.47 |
|  | GQ 01613 Plt 0493 | 77.81 | 26.3 | 234.83 | 129.5 | 105.33 | 268.67 | 33.83 | 139.17 | 5.3 | 79.15 | 2743.93 | 17.96 | 0.49 |
|  |  |  |  |  |  |  |  |  |  |  |  |  |  |  |
| 2 | GQ 00261 Plt 0885 | 76.6 | 26.8 | 249.67 | 165 | 84.67 | 322.67 | 73 | 157.67 | 5.9 | 78.3 | 1456.06 | 17.76 | 0.42 |
|  | GQ 00324 Plt 0993 | 77.75 | 25.2 | 224.67 | 139.5 | 85.17 | 285.33 | 60.67 | 145.83 | 5.7 | 79 | 1826.43 | 36.59 | 0.38 |
|  | GQ 00089 Plt 1143 | 76.89 | 25.5 | 185 | 112.83 | 72.17 | 234.17 | 49.17 | 121.33 | 5.5 | 78.3 | 2739.99 | 23.05 | 0.46 |
|  | GQ 00401 Plt 1167 | 77.17 | 23.9 | 225.17 | 161.08 | 64.08 | 305.33 | 80.17 | 144.25 | 5.9 | 78.3 | 2600.23 | 11 | 0.46 |
|  | GQ 00131 Plt 1414 | 78.45 | 25 | 193.75 | 130.17 | 63.58 | 276.25 | 82.5 | 146.08 | 5.9 | 80.65 | 1659.06 | 14.48 | 0.34 |
|  |  |  |  |  |  |  |  |  |  |  |  |  |  |  |
| 3 | GQ 01527 Plt 0106 | 78.24 | 22.7 | 243.83 | 139.58 | 104.25 | 272.58 | 28.75 | 133 | 5.8 | 79.8 | 1832.63 | 43.78 | 0.44 |
|  | GQ 01523 Plt 0218 | 77.58 | 20.5 | 239.08 | 146.92 | 92.17 | 283.25 | 44.17 | 136.33 | 5.87 | 78.3 | 1504.38 | 28.47 | 0.41 |
|  | GQ 01696 Plt 0376 | 78.65 | 19.4 | 201.25 | 114.67 | 86.58 | 238.08 | 36.83 | 123.42 | 5.9 | 79.8 | 1678.02 | 59.43 | 0.45 |
|  | GQ 01659 Plt 0492 | 78.43 | 19.9 | 265.58 | 138.33 | 127.25 | 259.17 | -6.42 | 120.83 | 5.93 | 80.6 | 2490.7 | 85.94 | 0.47 |
|  | GQ 01691 Plt 0543 | 79.94 | 22.7 | 283.58 | 164.17 | 119.42 | 288.67 | 5.08 | 124.5 | 6.1 | 83.05 | 1500.44 | 60.28 | 0.41 |

Table S2 (cont’d)

| Cluster | Sample | SPR | SMMAX (kPA) | LM_SMMAX (kPA) | TD_SMMAX | TEMP_GELPT (ºC) | TEMP_SMMAX (ºC) | TROUGH_SM (kPa) | SLOPE1_SM | SLOPE2_SM |
| --- | --- | --- | --- | --- | --- | --- | --- | --- | --- | --- |
| 1 | GQ 00403 Plt 0057 | 0.12 | 35453.33 | 2186.33 | 0.06 | 54.13 | 84.13 | 14166.67 | 10755.7225 | 1777.855 |
|  | GQ 01652 Plt 0222 | 0.1 | 33333.33 | 2676.33 | 0.08 | 59.5 | 84.77 | 15533.33 | 9101.285 | 1530.4575 |
|  | GQ 01524 Plt 0369 | 0.12 | 33410 | 3615 | 0.11 | 62.4 | 80.9 | 12750 | 9850.5 | 1329.95 |
|  | GQ 01633 Plt 0370 | 0.14 | 46170 | 4504 | 0.1 | 59.93 | 81.3 | 17850 | 4774.585 | 1674.375 |
|  | GQ 01613 Plt 0493 | 0.12 | 36256.67 | 4203.67 | 0.12 | 62.6 | 78.17 | 10926.67 | 7992 | 1894.3925 |
|  |  |  |  |  |  |  |  |  |  |  |
| 2 | GQ 00261 Plt 0885 | 0.12 | 26360 | 2250.33 | 0.09 | 59.03 | 89.4 | 14100 | 5444.6875 | 1755.2775 |
|  | GQ 00324 Plt 0993 | 0.11 | 37003.33 | 3567.33 | 0.1 | 56.37 | 80.2 | 13250 | 5357.8825 | 1390.9375 |
|  | GQ 00089 Plt 1143 | 0.1 | 19263.33 | 3796.33 | 0.21 | 59.03 | 84.83 | 12850 | 7788.0425 | 1486.7275 |
|  | GQ 00401 Plt 1167 | 0.11 | 15493.33 | 1619.33 | 0.1 | 58.13 | 90.93 | 9530 | 4268.9175 | 1016.285 |
|  | GQ 00131 Plt 1414 | 0.11 | 15206.67 | 2812.33 | 0.18 | 65.07 | 82.47 | 8650 | 5332.8525 | 1038.1975 |
|  |  |  |  |  |  |  |  |  |  |  |
| 3 | GQ 01527 Plt 0106 | 0.1 | 31160 | 3676.33 | 0.12 | 59.23 | 80 | 10250 | 3830.02 | 1291.42 |
|  | GQ 01523 Plt 0218 | 0.09 | 40073.33 | 4205.33 | 0.1 | 60.83 | 77.9 | 13033.33 | 11064.1675 | 1599.1725 |
|  | GQ 01696 Plt 0376 | 0.08 | 33356.67 | 3457.33 | 0.1 | 61.03 | 81.57 | 13666.67 | 8006.845 | 1478.68 |
|  | GQ 01659 Plt 0492 | 0.09 | 27200 | 4003.67 | 0.16 | 61.27 | 78.37 | 10165 | 8131.6025 | 1357.3625 |
|  | GQ 01691 Plt 0543 | 0.11 | 29146.67 | 2891 | 0.1 | 62.17 | 81.97 | 10723.33 | 4697.2225 | 1236.005 |

Table S2 (cont’d)

| Cluster | Sample | SLOPE3_LM | SLOPE4_LM | PC (%) |
| --- | --- | --- | --- | --- |
| 1 | GQ 00403 Plt 0057 | 2085.61 | 2655.8 | 8.27 |
|  | GQ 01652 Plt 0222 | 2162.9675 | 1899.445 | 8.03 |
|  | GQ 01524 Plt 0369 | 2869.25 | 3338.575 | 6.55 |
|  | GQ 01633 Plt 0370 | 2023.785 | 2472.99 | 6.84 |
|  | GQ 01613 Plt 0493 | 1450.97 | 673.57 | 7.74 |
|  |  |  |  |  |
| 2 | GQ 00261 Plt 0885 | 1312.4775 | 1300.4175 | 9.4 |
|  | GQ 00324 Plt 0993 | 631.4075 | 704.595 | 8.63 |
|  | GQ 00089 Plt 1143 | 2072.5 | 2499 | 8.33 |
|  | GQ 00401 Plt 1167 | 500.1975 | 614.5125 | 11.13 |
|  | GQ 00131 Plt 1414 | 1070.4 | 758.75 | 7.38 |
|  |  |  |  |  |
| 3 | GQ 01527 Plt 0106 | 1696.5 | 1155.4175 | 7.74 |
|  | GQ 01523 Plt 0218 | 1112.68 | 613.18 | 7.91 |
|  | GQ 01696 Plt 0376 | 1857.8325 | 2322.555 | 8.63 |
|  | GQ 01659 Plt 0492 | 539.5 | 2153.59 | 8.33 |
|  | GQ 01691 Plt 0543 | 864.1675 | 1757.4325 | 6.49 |

Table S3. Sensory evaluation scores^1^ for the fifteen rice accessions from the three clusters.

| Cluster | Sample | COH | COH_MASS | HRD | ISC | MOIST_ABS | RLP | ROUGH | SLICK | SPR | STK_GRAINS | STK_LIPS | TPK | UOB |
| --- | --- | --- | --- | --- | --- | --- | --- | --- | --- | --- | --- | --- | --- | --- |
| 1 | GQ 00403 Plt 0057 | 56.30 | 85.13 | 50.59 | 60.73 | 54.93 | 64.00 | 37.11 | 52.80 | 52.07 | 89.69 | 54.64 | 57.88 | 88.69 |
|  | GQ 01652 Plt 0222 | 71.67 | 94.44 | 52.89 | 59.56 | 52.50 | 66.00 | 41.22 | 77.89 | 52.89 | 55.56 | 72.33 | 69.00 | 86.33 |
|  | GQ 01524 Plt 0369 | 69.11 | 98.11 | 58.11 | 51.89 | 85.44 | 77.89 | 55.00 | 78.67 | 65.00 | 93.56 | 75.67 | 70.11 | 92.67 |
|  | GQ 01633 Plt 0370 | 78.78 | 97.11 | 56.44 | 65.78 | 67.78 | 69.78 | 44.56 | 75.44 | 55.67 | 63.11 | 99.00 | 79.11 | 96.89 |
|  | GQ 01613 Plt 0493 | 73.78 | 91.67 | 62.67 | 83.56 | 66.67 | 66.89 | 50.33 | 49.22 | 69.22 | 80.89 | 82.00 | 81.44 | 89.67 |
|  |  |  |  |  |  |  |  |  |  |  |  |  |  |  |
| 2 | GQ 00261 Plt 0885 | 38.88 | 74.99 | 44.33 | 53.30 | 61.34 | 67.92 | 35.03 | 62.37 | 38.43 | 57.53 | 67.86 | 48.39 | 105.01 |
|  | GQ 00324 Plt 0993 | 65.78 | 73.33 | 61.33 | 54.67 | 73.56 | 74.89 | 44.11 | 62.11 | 47.56 | 69.44 | 79.11 | 76.44 | 82.56 |
|  | GQ 00089 Plt 1143 | 43.78 | 66.78 | 48.67 | 60.44 | 57.11 | 82.11 | 45.44 | 59.89 | 48.56 | 57.78 | 48.89 | 42.44 | 76.67 |
|  | GQ 00401 Plt 1167 | 47.29 | 49.79 | 53.28 | 51.35 | 67.76 | 85.76 | 66.38 | 51.45 | 52.99 | 35.62 | 47.65 | 37.46 | 78.63 |
|  | GQ 00131 Plt 1414 | 51.26 | 64.71 | 51.03 | 47.47 | 63.91 | 65.06 | 59.89 | 43.22 | 59.08 | 56.44 | 49.54 | 51.15 | 72.64 |
|  |  |  |  |  |  |  |  |  |  |  |  |  |  |  |
| 3 | GQ 01527 Plt 0106 | 65.92 | 86.31 | 54.77 | 63.38 | 76.62 | 50.31 | 44.85 | 52.92 | 49.38 | 84.92 | 97.85 | 56.15 | 106.15 |
|  | GQ 01523 Plt 0218 | 72.55 | 85.09 | 48.55 | 67.55 | 72.27 | 60.27 | 40.73 | 66.09 | 59.00 | 75.64 | 98.09 | 69.73 | 103.55 |
|  | GQ 01696 Plt 0376 | 56.17 | 84.33 | 38.58 | 76.67 | 75.83 | 68.17 | 50.75 | 82.50 | 56.83 | 82.42 | 102.25 | 73.42 | 101.83 |
|  | GQ 01659 Plt 0492 | 74.00 | 107.11 | 42.00 | 77.56 | 70.00 | 39.33 | 36.00 | 61.78 | 52.22 | 82.00 | 104.33 | 71.00 | 115.78 |
|  | GQ 01691 Plt 0543 | 71.56 | 95.11 | 33.78 | 76.33 | 57.78 | 60.22 | 31.56 | 83.33 | 58.56 | 87.44 | 95.67 | 61.89 | 111.78 |

^1^ The attributes evaluated by the sensory panel were: cohesiveness (COH), cohesiveness of mass (COH_MASS), hardness (HRD), initial starchy coating (ISC), moisture absorption (MOIST_ABS), residual loose particles (RLP), roughness (ROUGH), slickness (SLICK), springiness (SPR), stickiness between grains (STK_GRAINS), stickiness to the lips (STK_LIPS), toothpack (TPK), uniformity of bite (UOB)
